# How Online Basic Psychological Need Satisfaction Influences Self-Disclosure Online among Chinese Adolescents: Moderated Mediation Effect of Exhibitionism and Narcissism

**DOI:** 10.3389/fpsyg.2016.01279

**Published:** 2016-08-26

**Authors:** Ying Liu, Ru-De Liu, Yi Ding, Jia Wang, Rui Zhen, Le Xu

**Affiliations:** ^1^Beijing Key Laboratory of Applied Experimental Psychology, School of Psychology, Beijing Normal University, BeijingChina; ^2^Graduate School of Education, Fordham University, New York, NYUSA

**Keywords:** online basic psychological need satisfaction, exhibitionism, narcissism, self-disclosure, Chinese adolescents

## Abstract

Under the basic framework of self-determination theory, the present study examined a moderated mediation model in which exhibitionism mediated the relationship between online basic psychological need satisfaction and self-disclosure on the mobile Internet, and this mediation effect was moderated by narcissism. A total of 296 Chinese middle school students participated in this research. The results revealed that exhibitionism fully mediated the association between online competence need satisfaction and self-disclosure on the mobile net, and partly mediated the association between online relatedness need satisfaction and self-disclosure on the mobile net. The mediating path from online basic psychological need satisfaction (competence and relatedness) to exhibitionism was moderated by narcissism. Compared to the low level of narcissism, online competence need satisfaction had a stronger predictive power on exhibitionism under the high level of narcissism condition. In contrast, online relatedness need satisfaction had a weaker predictive power on exhibitionism.

## Introduction

With the rapid development and popularity of smart phone technology, people increasingly enjoy accessing to the Internet through mobile phones (Ishii, 2004; Wu et al., 2013; Wells et al., 2014), especially for adolescents (Hollenbaugh, 2011; Wang J.L. et al., 2011). The smart phone provides optimal conditions for people to access social networking sites (SNS) anytime and anywhere with its characteristics of portability and convenience. The existing research has indicated that using SNS through mobile device can increase self-disclosure behavior (Kwak et al., 2014). Online self-disclosure represents the amount of information that an Internet user intends to reveal to others (Joinson and Paine, 2007). It includes two dimensions, namely, breadth and depth (Levinger and Snoek, 1972; Wheeless, 1978; Hollenbaugh and Ferris, 2014). Breadth refers to the diversity of topics involved in the process of disclosing online. Depth is characterized by more personal or intimate disclosures. Through SNS such as Facebook (Kisekka et al., 2013; Kim, 2016), blogs (Hollenbaugh, 2010; Chen, 2012), WeChat (Wen, 2014), and Twitter (Choi and Toma, 2014; Klausen, 2015) on the mobile net, users can disclose particular information as they talk about various topics, communicate deeply, post photos or videos, and update their status. The previous research on online self-disclosure can be divided into three categories: comparison between online self-disclosure and offline self-disclosure (Nguyen et al., 2012; Emanuel et al., 2014); the influence factors of online self-disclosure (Valkenburg and Peter, 2007; Rosen et al., 2010; Kisilevich and Last, 2011; Forest and Wood, 2012; Hollenbaugh and Ferris, 2014); and the effect of online self-disclosure (Joinson et al., 2007; Bryce and Klang, 2009; Ko and Kuo, 2009; Zimmer et al., 2010; Kisilevich and Last, 2011; Park et al., 2011).

Self-disclosure online has both positive and negative consequences. Basic psychological need satisfaction is one of the important positive effects of self-disclosure online (Smock et al., 2011; Ang et al., 2015). In specific, self-disclosure online can produce psychological benefits such as a sense of autonomy, recognition, and belongingness. Previous studies have concentrated on the gratifications of various Internet usages, but few studies focused on the impact of gratifications from Internet usage on human network behavior. In particularly, compared to adults, teenagers prefer to disclose more private information through the Internet (McKenna et al., 2002; Gibbs et al., 2006; Joinson and Paine, 2007; Mazer et al., 2007; Walther, 2011), especially at the age of 15 years (Ma and Leung, 2006; Valkenburg and Peter, 2007). Thus, the assumption comes, is the satisfied psychological need in the network the reason why teenagers are more likely to disclose on the Internet? Therefore, the present study intended to investigate the effect of online basic psychological need satisfaction on self-disclosure on the mobile net among adolescents. Furthermore, it is of great value to futher explore the underlying mechanism such as the mediator or moderator of the association between online basic psychological need satisfaction and self-disclosure to reveal valuable information about the underlying processes through which this relationship occurs.

Psychological needs are the fundamental driving force of an individual’s behavior. Self-determination theory (SDT) provides a unique perspective to understand the relationship between psychological needs and network behavior (Zhu et al., 2011; Shen et al., 2013). According to SDT, there are three basic psychological needs: competence refers to the need to perform successful social interactions with skills and ability; relatedness considers one’s need to feel connected with others; and autonomy refers to the need to decide one’s own behavior and act freely (Deci and Ryan, 1985). The purpose of various human behaviors is to satisfy the basic psychological needs throughout the lifetime (Deci and Ryan, 2000). Self-disclosure online such as publishing opinions, posting photos or videos, and communicating with other users through social media affords disclosers the opportunities to satisfy basic psychological needs (Ang et al., 2015). Besides, people become more or less interested in those activities as a function of the degree to which they experience satisfaction of competence, relatedness, and autonomy while engaging in the activities (Deci and Ryan, 2000). That is, the fulfilled basic psychological needs provide positive feedback on network behavior. The higher the need being satisfied in the activity, the stronger the internal motivation of the activity and the more behavior involved (Moller et al., 2010). Based on the above theories, the current study concluded that the more satisfaction experienced online, the more engagement in online disclosure behavior.

A series of empirical research studies on network activities, such as behavior in the network learning environment (Chen and Jang, 2010), Internet usage (Zhao et al., 2011), network games (Ryan et al., 2006; Przybylski et al., 2009; Wang C.K.J. et al., 2011), and Facebook usage (Sheldon and Gunz, 2009; Sheldon et al., 2011) showed that the higher the degree of satisfaction of psychological needs, the more the online behavioral involvement. For example, a research on online games indicated that children who perceived higher level of satisfaction of competence, relatedness and autonomy need online tended to use the Internet more often (Shen et al., 2013). Thereby, the existing studies indirectly demonstrated that the inherent properties of the experiences provided by the Internet motivated children’s sustained Internet engagement. It was inferred that the rewarding experience of obtaining these gratifications online, in turn, might become compulsive and cause increasingly more Internet usages to satisfy the same needs repeatedly. However, the previous studies mainly focused on children or other network behaviors, few empirical studies have directly investigated the relationship between online basic psychological need satisfaction and self-disclosure on the mobile net among adolescents. Adolescence is a critical period of rapid physical and mental growth and development, during which adolescents are not only eager to be free from parents and teachers’ control, but are also eager to obtain understanding and admiration from others (Fuligni, 1998). To extend the literature, this research aimed to examine the influence of online basic psychological need satisfaction on self-disclosure on the mobile net among adolescents.

Moreover, motivation is derived from the need to act directly on the behavior. Accordingly, motivation acts as a mediator between need and behavior. In the virtual context, psychological and social variables affect motives for media use, which in turn predict the frequency and type of media use (Katz et al., 1974; Rubin, 2002). Exhibitionism is a critical motivation for users to create content online (Koskela, 2004; Hollenbaugh, 2011; Hollenbaugh and Ferris, 2014), which deserves further explanation (Hollenbaugh and Ferris, 2015). Exhibitionism is considered as the desire of individuals to frequently present their private lives in public to attract other’s attention (Koskela, 2004). It includes a combination of self-display, vanity, and superiority. People who score higher in exhibitionism tend to demand more social attention and reveal self-promoting information online to attract attention and appreciation from others. The prevalence of mobile phones and social media like Facebook and blogs has further promoted exhibitionism (Koskela, 2004; Qian and Scott, 2007; Schmidt, 2007). It has been demonstrated that exhibitionism positively predicted self-disclosure (Hollenbaugh, 2011; Ryan and Xenos, 2011; Hollenbaugh and Ferris, 2014, 2015).

Theoretical and empirical studies indicated that adolescents’ online basic psychological need satisfaction can promote exhibitionism, which in turn predicts self-disclosure online. According to SDT, basic psychological need satisfaction is the internal dynamics of behavior, which enhances internal motivation and facilitates the internalization of external motivation (Deci and Ryan, 2000). With the development of independent consciousness and self-consciousness, adolescents increasingly show a strong desire for self-presentation in adolescence (Mazur, 2010). They are eager to exhibit their abilities and skills to maintain their self-image and obtain the recognition and appreciation from others. According to Bandura, feelings of personal competence are related to self-perceptions of efficacy regarding one’s ability in dealing with distinct social domains, and are seen as the proximal and direct predictors of psychological motivation (Bandura et al., 1999). Internet provides an excellent opportunity for adolescents to experience the feeling to be unique and good at certain skills. In the virtual context, individuals are more willing to engage in exhibition when obtaining higher satisfied competence need. Similarly, exhibitionism motivation usually exists in interpersonal interaction. To satisfy relatedness need, people always spend a lot of efforts to get the appreciation and affection from others. In order to obtain close relationships, people are concerned more about their performance and self-image (McClelland, 1976). The background behind exhibiting is to leave a good impression on others. The mobile phone network plays an active role in social development, such as its role in promoting the exchange of information and expanding the scope of students’ interpersonal communication. In the virtual context, relatedness need motivates people to maintain positive self-image through self-expression and online display (Gibbs et al., 2006). When individuals experience the caring, understanding and support from the surrounding environment or other people online, people usually tend to have more positive self-expression. In addition, when individuals feel they can decide their own behavior online, they experience a kind of internal attribution, then the internal motivation of participating in the activities is high. In short, satisfied psychological needs can facilitate the internalization of external motivation and promote the individual to insist on a certain activity over a period of time.

The relation between basic psychological need satisfaction and exhibitionism is also supported by some empirical research. Hollenbaugh (2011) posited that recognition (competence) and social (relatedness) needs underlie the motivations for producers of Internet content. Relatedness and competence needs can be satisfied by being overly exhibitionistic (Lee and Robbins, 1995). Strong feelings of satisfaction should be positively reinforced and act as a motivational force for more behavioral involvement (Moller et al., 2010).

In conclusion, this study proposed a mediation model, demonstrating that exhibitionism mediated the relationship between online basic psychological need satisfaction and self-disclosure on the mobile net. Notably, much empirical work posited that competence, relatedness, and autonomy need satisfaction each made unique predictive contributions to human behavior (Sheldon and Gunz, 2009). Therefor, we formulated the following hypotheses to, respectively, investigate the impact of three kinds of basic psychological need satisfaction on self-disclosure on the mobile net (H1–H3) and the mediating role of exhibitionism among the associations (H4):

(i) **H1** Competence need satisfaction positively predicts self-disclosure on the mobile net.
(ii) **H2** Relatedness need satisfaction positively predicts self-disclosure on the mobile net.
(iii) **H3** Autonomy need satisfaction positively predicts self-disclosure on the mobile net.
(iv) **H4** Exhibitionism mediates the associations between online psychological need satisfaction (competence, relatedness, autonomy) and self-disclosure on the mobile net.

The mediation effect model focuses on the influence mechanism of the independent variable on the dependent variable. Nevertheless, this approach cannot answer the question of when the influence power will be more effective. In fact, the degree of basic psychological need satisfaction varies with each individual, and this difference would be reflected in the motivation of online behavior. Research has showed that personality was an important internal cause of the individual differences for need satisfaction and motivation (Caspi et al., 2005). Personality moderated the impact of individuals’ need on motivation, which would lead to the individual differences in motivation (Leary, 1999). Therefore, this study proposed that exhibitionism was not only influenced by online basic psychological need satisfaction, but was also influenced by individuals’ personality traits.

Narcissism is one of the personality factors that influences the exhibitionism (Wang and Stefanone, 2013). The social-personality perspective conceptualizes narcissism as a long-term, diversified, and comprehensive personality trait that is not necessarily pathological (Morf and Rhodewalt, 2001; Campbell et al., 2006). It is commonly found in individuals (Miller and Campbell, 2010). Indeed, narcissism may be adaptive in some ways (Sedikides et al., 2004). Narcissists usually have positive self-concept, and they will view themselves in a positive way. The main characteristics of narcissism include grandiosity, positive self-evaluation, self-importance, lack of empathy, and a need for admiration (Wink, 1991; Campbell, 1999; Morf and Rhodewalt, 2001; Campbell and Foster, 2002; Ames et al., 2006). Stemming from the underlying need to exhibit superiority, narcissism positively predicts exhibitionism (Wink, 1991; Morf and Rhodewalt, 2001). A narcissist who has a strong desire to be admired by others is associated with higher exhibitionism motivation to disclose private information that emphasizes attractiveness.

Actually, it is even claimed that social media such as Facebook, blogs, and Twitter specifically provides a platform for narcissistic individual to fulfill the basic psychological need by exhibiting superiority. Previous studies have indicated that the degree of need satisfaction from using Facebook differs as a function of personality (Park et al., 2009; Ross et al., 2009; Urista et al., 2009). Narcissistic individuals themselves have a very strong exhibitionism motivation, which drives them to exhibit their talents to others to get attention and admiration. Thus, the easy accessibility of the smart phone gratifies the narcissistic individuals’ need to engage in self-promotion that ultimately reveals his or her exhibitionist tendencies. Narcissists are gratified largely by the exhibitionistic nature of SNS (Bibby, 2008; Wang and Stefanone, 2013). This implies that people with high level of narcissism have a stronger exhibitionism motivation when their psychological needs are gratified online.

In addition, narcissism increases significantly between the ages of 14 and 18 years (Carlson and Gjerde, 2009). Teenagers of about 15 years old are in the center stage of puberty, and they particularly desire capability, support and autonomy. Therefore, considering personality characteristics is helpful to understand the effect of basic psychological need satisfaction of Internet users with different personality types on self-disclosure on the mobile net. In summary, we proposed the following hypothesis:

(i) **H5** Narcissism moderates the relationship between online basic psychological need satisfaction (competence, relatedness, autonomy) and exhibitionism.

## Materials and Methods

### Participants

The random sampling method was used to select 296 middle school students (females: 175, males: 121; average age = 16.90 years, standard deviation = 1.36) from an ordinary middle school in Beijing city to participate in the survey. All participants were assured that their responses would be kept confidential and irrelevant to their course grade. After completing the questionnaires, participants received course credit. All the participants in the survey were smart phone users, and all had experience with accessing to the Internet through smart phones.

### Ethical Statement

The local ethical committee of Beijing Normal University approved this study. Written informed consent was obtained from school principal, teachers and parents of all of these students. All participants were informed of their right to withdraw from the survey at any time.

### Measures

#### Online Basic Psychological Need Satisfaction

Participants’ online basic psychological need satisfaction was measured by an online need satisfaction questionnaire (Shen et al., 2013). In order to ensure the domain specific measure, each item was associated with a mobile Internet situation. The scale included three subscales: autonomy (four items, e.g., I felt a certain freedom of action when I used the Internet by mobile phone), competence (four items, e.g., I am satisfied with my performance on mobile Internet) and relatedness (four items, e.g., When I was on mobile Internet, I feel I was supported by others online). A 7-point scale was provided, ranging from 1 (*strongly disagree*) to 7 (*strongly agree*). Higher scores indicated higher basic psychological need satisfaction perceived online. A reliability test based on the data of this study revealed a satisfactory internal consistency (α = 0.824, 0.798, 0.888 for autonomy, competence, and relatedness, respectively).

#### Exhibitionism

For assessing participants’ tendency toward exhibitionism when using the mobile Internet, five items from the subscale of a Facebook motives questionnaire were chosen for this study (Hollenbaugh and Ferris, 2014). In order to ensure the domain specific measure, each item was associated with a mobile net situation. An additional item was created to measure participants’ desire to use the mobile Internet to get somebody’s attention. The final measure consisted of six items (The new item: If no one can see, I’m not going to publish content on the mobile net). Each item was answered using a 5-point scale ranging from 1 (*strongly disagree*) to 5 (*strongly agree*). The confirmatory factor analysis was carried out, and the overall fitting index of the scale was as follows: *χ^2^/df* = 2.325, CFI = 0.988, TLI = 0.973, RMSEA = 0.067. A reliability test based on the data of this study revealed a satisfactory internal consistency (α = 0.838). The analysis results showed that the exhibitionism scale had good reliability and construct validity.

#### Narcissism

Narcissism was assessed using the revised 16-item Narcissism Personality Inventory (NPI-16; Ames et al., 2006; Jones and Paulhus, 2014). According to previous studies (Penney and Spector, 2002; Aviram and Amichai-Hamburger, 2005; Leung, 2013), participants responded on a 5-point scale ranging from 1 (*strongly disagree*) to 5 (*strongly agree*) in the current study. The internal reliability coefficient for narcissism was 0.70.

#### Self-Disclosure on the Mobile Net

For assessing self-disclosure on the mobile net, a slightly revised version of the self-disclosure online scale was adapted from previous studies (Valkenburg and Peter, 2007; Wang J.L. et al., 2011). These scales consisted of two dimensions. Each dimension contained four items to assess both breadth and depth of self-disclosure on the mobile net (We deleted an item from the depth dimension that was related to sex and was not suitable for young people.). In order to ensure the domain specific measure, each item was associated with a mobile net situation. Participants responded on a 5-point scale ranging from 1 (*strongly disagree*) to 5 (*strongly agree*). In this study, the internal reliability coefficients for breadth and depth were 0.851 and 0.852.

Finally, basic demographic information was requested, including gender and age.

### Procedure and Data Analysis

The investigation was conducted in the students’ classroom. Trained research assistants were present during the entire process. Research assistants instructed the participants to express their personal opinions and judgments before answering the questionnaires. The participants did not need to write their names on the questionnaires, and the confidentiality of their responses was assured. The basic psychological need satisfaction online questionnaire was administered first, followed by the exhibitionism scale, the narcissism scale and the self-disclosure on the mobile net scale. All the questionnaires administrated in this study were in the Chinese language. It took approximately 40 min for the students to complete all the instruments.

Data were collected through a series of questionnaires. First, descriptive statistics were calculated to characterize the sample, including information about students’ gender and age. Second, correlations among the seven main variables (independent variables: competence, relatedness, autonomy; mediator variable: exhibitionism; moderator variable: narcissism; dependent variables: breadth, depth) were also analyzed. Furthermore, a structural equation modeling (SEM; Joreskog and Sorbom, 1979) analysis was conducted in order to explore the hypothesized model by AMOS 17.0 in two interlinked steps. In the first step, we tested the mediation models (Hypotheses 1–4). A bootstrap estimation procedure with 1000 bootstrap samples was used to test the significance of mediation effects. In the second step, we integrated the proposed moderator variable into the model and empirically examined the overall moderated mediation (Hypothesis 5).

## Results

### Preliminary Analyses

In this study, data were collected by means of self-report questionnaires (**Supplementary Data Sheets 1** and 2). Harman’s single factor test method was adopted to examine the common method bias (Podsakoff et al., 2003). Results showed that the first un-rotated factor explained the variance of 27.739%, far less than the critical value. Therefore, the influence of common method bias in this study was not serious.

Descriptive statistics for all variables are showed in **Table 1**. In addition, difference in disclosure online by gender was analyzed. Independent-samples *t*-test demonstrated that there was no significant difference between girls and boys in breadth and depth of self-disclosure on the mobile net (*t* = 0.042, *p* > 0.05; *t* = 0.041, *p* > 0.05). Results of correlations analysis indicated that self-disclosure on the mobile net was in various degrees significantly associated with all six variables. Satisfaction of all three online needs satisfaction was significantly correlated with breadth (*r* ranged from 0.37 to 0.45; *p* < 0.01) and depth of self-disclosure on the mobile net (*r* ranged from 0.38 to 0.55; *p* < 0.01). Exhibitionism was significantly positively correlated with satisfaction of three needs (*r* = 0.27, 0.43, 0.46, *p* < 0.01) and self-disclosure (*r* = 0.31, 0.41, *p* < 0.01). Autonomy, competence, and relatedness needs satisfaction and narcissism had significant but weak positive correlation (*r* = 0.19, 0.27, 0.27, *p* < 0.01), indicating that the independent variable and moderate variable had relative independence with each other, suitable for a subsequent moderating effect analysis. The hypothesis model of this research can be further analyzed.

**Table 1:** Descriptive statistics and correlations among the variables. (*N* = 296).

Variables	*M*	*SD*	1	2	3	4	5	6
1. Autonomy	4.73	1.26	–					
2. Competence	3.90	1.27	0.64**	–				
3. Relatedness	3.75	1.36	0.56**	0.79**	–			
4. Narcissism	3.00	0.45	0.19**	0.27**	0.27**	–		
5. Exhibitionism	2.97	0.85	0.27**	0.43**	0.46**	0.20**	–	
6. Breadth	3.15	0.92	0.39**	0.37**	0.45**	0.26**	0.31**	–
7. Depth	2.89	1.02	0.38**	0.43**	0.55**	0.23**	0.41**	0.70**

*p < 0.05; **p < 0.01.

### The Effect of Online Basic Psychological Need Satisfaction on Self-Disclosure on the Mobile Net: The Mediating Effect of Exhibitionism

**FIGURE 1:**
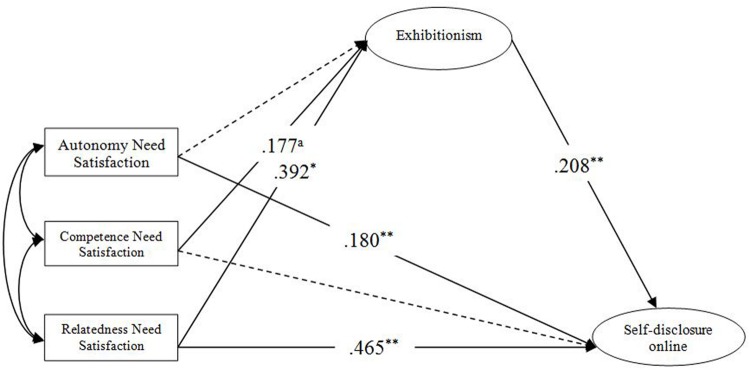
**Finalized structural model.** ^a^Represents: marginally significant. *p < 0.05, **p < 0.01.

According to the process of the moderated mediation model test procedure, this study first examined the mediating effect of exhibitionism, and then examined the moderating effect of narcissism. Structural equation modeling (Joreskog and Sorbom, 1979) by AMOS 17.0 was performed to test our hypothesized mediation model. The results of the final model (**Figure 1**) indicated a good fit with the data: *χ2/df* = 2.605, *p* < 0.001; IFI = 0.964; TLI = 0.945; CFI = 0.964; and RMSEA = 0.074. As we hypothesized, online autonomy and relatedness needs satisfaction positively predicted self-disclosure on the mobile net (β = 0.18, *p* < 0.01, β = 0.47, *p* < 0.01), partially providing support for H1–H3. Specifically, online competence and relatedness needs satisfaction positively predicted exhibitionism (β = 0.18, *p* = 0.07, β = 0.39, *p* < 0.05). In turn, exhibitionism positively predicted self-disclosure on the mobile net (β = 0.21, *p* < 0.01). Further analysis found that exhibitionism showed a full mediation effect on the path from online competence need satisfaction to self-disclosure on the mobile net, and a partial mediation effect on the path from online relatedness need satisfaction to self-disclosure on the mobile net. There was no mediating effect of exhibitionism between online autonomy satisfaction and self-disclosure on the mobile net. In the present study, a bootstrapping method with 1000 bootstrap samples was used to test the significance of mediation effects. If the CIs did not include zero (*p* < 0.05), we concluded that the mediated effects were statistically significant (Preacher and Hayes, 2008). Bootstrap analysis testified that the indirect effect of online competence need satisfaction on self-disclosure on the mobile net via exhibitionism was significantly different from zero (90% CI = 0.002–0.056). The indirect effect of online relatedness need satisfaction on self-disclosure on the mobile net via exhibitionism was significantly different from zero (90% CI = 0.015–0.098). Therefore, H4 was partially supported.

### The Moderate Effect of Narcissism

**FIGURE 2:**
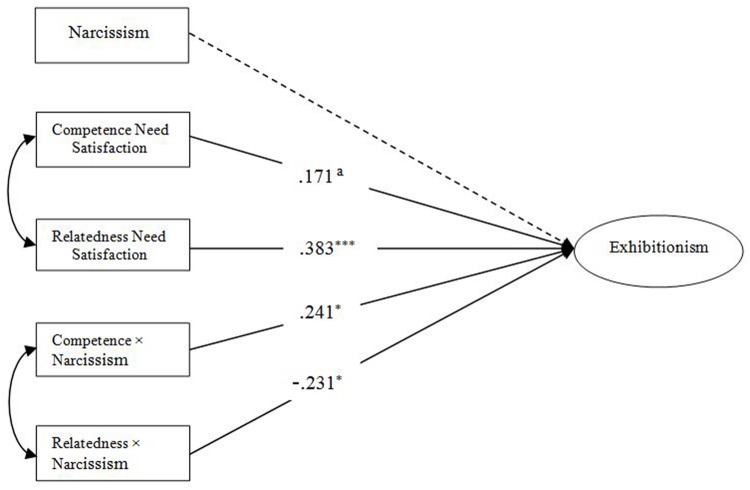
**Finalized structural model.** ^a^Represents: marginally significant. *p < 0.05, ***p < 0.001.

In this part of the present study, the independent variables were online competence and relatedness needs satisfaction, the moderator variable was narcissism, and the dependent variable was exhibitionism. Because there was no mediating effect of exhibitionism between online autonomy satisfaction and self-disclosure on the mobile net, the independent variable did not include online autonomy satisfaction in this part of the analysis. Centralizing the independent variables and moderator variable, we investigated the interaction effect of both online competence and relatedness needs satisfaction and narcissism on exhibitionism using structural equation model. The results of the final model (**Figure 2**) indicated a good fit with the data: χ^2^*/df* = 3.307, *p* < 0.001; IFI = 0.938; TLI = 0.918; CFI = 0.938; and RMSEA = 0.088. As we hypothesized, online competence and relatedness needs satisfaction significantly positive predicted exhibitionism (β = 0.17, *p* = 0.06; β = 0.38 *p* < 0.001), the predictive effect of narcissism was not significant. The interaction effect of online competence need satisfaction and narcissism significantly and positively predicted exhibitionism (β = 0.24, *p* < 0.05), while the interaction effect of relatedness need satisfaction and narcissism significantly and negatively predicted exhibitionism (β =–0.23, *p* < 0.05). Research results showed that narcissism played a significant positive moderating role in the influence of competence need satisfaction on exhibitionism. On the contrary, narcissism played a significant negative moderating role in the influence of relatedness need satisfaction on exhibitionism.

**FIGURE 3:**
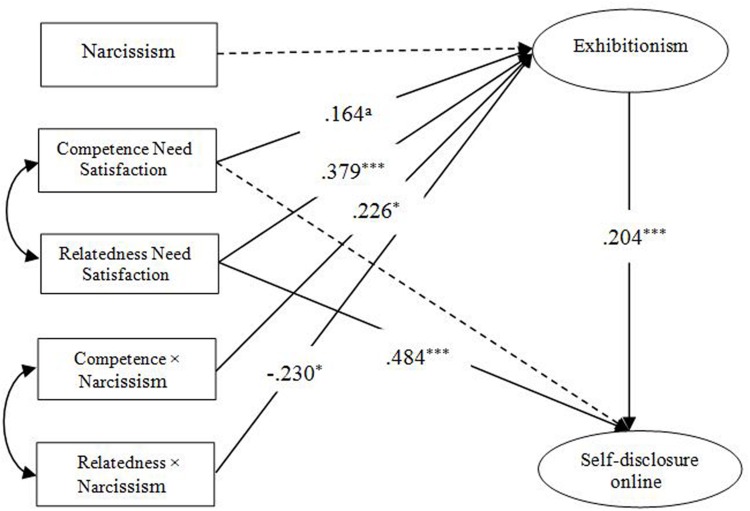
**Finalized structural model.** ^a^Represents: marginally significant. *p < 0.05, ***p < 0.001.

Based on the above considerations, this study validated the integration model. The result of the final model (**Figure 3**) indicated a good fit with the data: χ^2^*/df* = 2.810, *p* < 0.001; IFI = 0.943; TLI = 0.925; CFI = 0.942; and RMSEA = 0.078.

**FIGURE 4:**
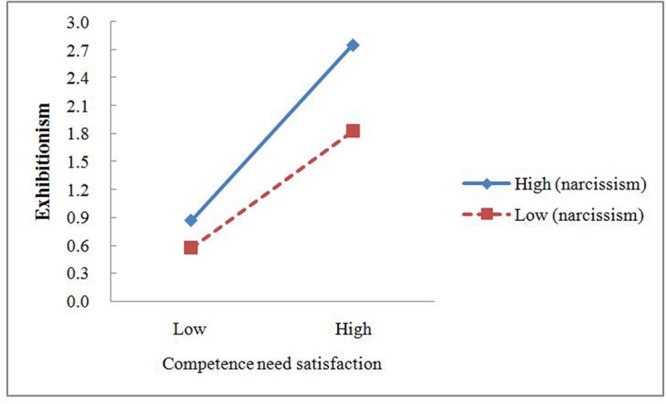
**The interactive effect of online competence need satisfaction and narcissism on exhibitionism**.

**FIGURE 5:**
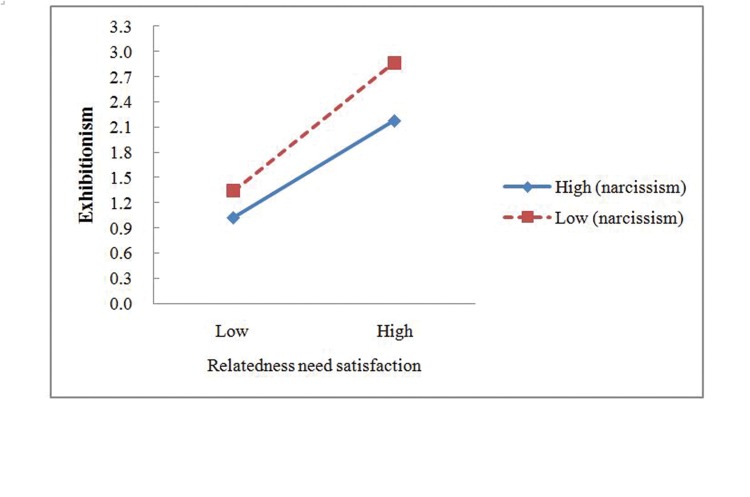
**The interactive effect of online relatedness need satisfaction and narcissism on exhibitionism**.

In order to reveal the essence of the interaction effect, this study used a simple slope analysis to analyze the specific moderating effect of narcissism. First, subjects were divided into two groups according their narcissism scores. One group represented high level of narcissism with scores that were higher than one standard deviation; the other group represented the low level of narcissism with scores that were lower than one standard deviation. Second, the linear regression was used, the independent variables were online competence or relatedness needs satisfaction, and the dependent variable was exhibitionism. Regression coefficients of the predictive effect of online competence need satisfaction on exhibitionism in the high level of narcissism group and in the low level of narcissism group were 0.47 (*t* = 6.73, *p* < 0.01) and 0.31 (*t* = 3.84, *p* < 0.01), respectively. Third, the same method was used to divide the participants into two groups, with one group scored higher than one standard deviation and the other group scored lower than one standard deviation on online competence need satisfaction. We calculated the average scores of the two groups on online competence need satisfaction and entered them into the two regression equations mentioned above. Then the exhibitionism scores under different conditions of narcissism and online competence need satisfaction were calculated, as shown in **Figure 4**. Under the higher level of narcissism condition, the promoting effect of online competence need satisfaction on exhibitionism was stronger than under the lower level of narcissism condition. In addition, regression coefficients of the predictive effect of relatedness need satisfaction on exhibitionism in the high level of narcissism and in the low level of narcissism group were 0.47 (*t* = 6.69, *p* < 0.01) and 0.39 (*t* = 4.84, *p* < 0.01). Next, participants were divided into two groups, with one group scored higher than one standard deviation and the other group scored lower than one standard deviation on online relatedness need satisfaction. We calculated the average scores of the two groups on online competence need satisfaction and entered them into the two regression equations. The exhibitionism scores under different conditions of narcissism and online relatedness need satisfaction were calculated, as shown in **Figure 5**. On the contray, under the lower level of narcissism condition, the promoting effect of online relatedness need satisfaction on exhibitionism was stronger. These findings validated the H5.

In summary, exhibitionism was proved to fully mediate the association between online competence need satisfaction and self-disclosure on the mobile net, but only partly mediated the association between online relatedness need satisfaction and self-disclosure on the mobile net. Moreover, narcissism moderated the mediation paths. Specifically, when the online competence need satisfaction was higher, adolescents with a high level of narcissism showed a stronger exhibitionism motivation than those with a low level of narcissism, while when the online relatedness need satisfaction was higher, the result was exactly opposite.

## Discussion

Based on the SDT, this study constructed a moderated mediation model to examine the mediation effect of exhibitionism in the relationship between online basic psychological need satisfaction and self-disclosure on the mobile net, and the moderating role of narcissism in the association between online basic psychological need satisfaction and exhibitionism. The results partially validated our research hypotheses. Exhibitionism was proved to fully mediate the association between online competence need satisfaction and self-disclosure on the mobile net, but only partly mediated the association between online relatedness need satisfaction and self-disclosure on the mobile net. Moreover, narcissism moderated the mediation paths from both competence and relatedness need satisfaction and exhibitionism. This research contributed to providing some theoretical and practical implications for practice and future research on self-disclosure online.

Inconsistent with previous research results (Winter et al., 2014), gender had no significant effect on self-disclosure on the mobile net in this study. Adolescents about 15 years old particularly enjoy accessing the Internet to disclose private information about themselves (Valkenburg and Peter, 2007). Regardless of gender, both boys and girls have a strong desire to satisfy their psychological need, so this study speculated that this may be one of the reasons why there was no gender difference.

### The Mediating Role of Exhibitionism in the Association between Online Basic Psychological Need Satisfaction (Competence and Relatedness) and Self-disclosure on the Mobile Net

Following the need-motivation-behavior model, this study introduced the important mediator variable of exhibitionism into the relationship between online basic psychological need satisfaction and self-disclosure on the mobile net. In line with our assumptions, the results of structural equation model showed that exhibitionism fully mediated the relationship between online competence need satisfaction and self-disclosure on the mobile net, and partially mediated that between online relatedness need satisfaction and self-disclosure on the mobile net. In addition, there was no significant mediation effect of exhibitionism in the relationship between online autonomy need satisfaction and self-disclosure on the mobile net.

Online competence need satisfaction did not directly promote the self-disclosure behavior of the young people in this study, and it completely depended on the mediating role of the exhibitionism motivation. This was most likely due to the physical and mental characteristics of the adolescents. Adolescence is a developmental stage at which individuals may experience a number of stressors including completing academic requirements, developing appropriate social roles with peers, and achieving expectations of increasing independence from family (Compas et al., 2001). At the core of this type of adolescent rebellion is the expression of the ego and the need for societal recognition. Most adolescents desire to be attractive and desirable. They have a strong need to express themselves and attract attention, and receive the recognition and approval of others through their own efforts around them. The advent of the Internet created a diversified open platform for students to exhibit their appearance, performance, and ability online. Correspondingly, the feeling of competence would further enhance their senses of recognition and affirmation, which encourage them to actively exhibit themselves which is in line with the exhibitionist desire of young boys and girls. According to the SDT, this shows a cyclical process in which teenagers with more online satisfaction actively seek opportunities for self-expression in order to get more attention and appreciation from others. Therefore, competence need satisfaction online leads to self-disclosure on the mobile net through exhibitionism.

Our findings indicated that the link between online relatedness need satisfaction and self-disclosure on the mobile net was significantly and partially mediated by exhibitionism. Adolescence is characterized by a struggle for individual independence. With the rapidly change of physiology and the psychological, adolescents are more likely to feel lonely specially and desire to be understood (Maes et al., 2015). Feelings of loneliness arise a signal to people that there is something missing in their social relationships and to motivate them to reconnect again. Although they have an urgent need to be related to and supported by peers other than their parents, it is difficult for them to show their real face in front of teachers and parents. The Internet has created virtual spaces and communities for young people to find a sense of belonging and to relieve their loneliness. Self-disclosure online is the act of making the self known to others, and it can serve to strengthen relationships. Through self-disclosure online, adolescents obtain more relatedness satisfaction. The online relatedness psychological need satisfaction reinforces subsequent network behavior to pursue more satisfaction. In addition, the link between online relatedness need satisfaction and self-disclosure on the mobile net was partially mediated by exhibitionism in this study. Actually, the motivation of self-disclosure online also includes seeking companionship, relationship maintenance, entertainment, and so on (Hollenbaugh and Ferris, 2014). Besides, with the stability and deepening of the relationship, maybe other motivations like relationship maintenance should be included in the future study.

In addition, our findings indicated that the link between online autonomy need satisfaction and self-disclosure on the mobile net was not mediated by exhibitionism. Teenagers try to become independent individuals on a psychological level and become free of dependence on their parents. But in real life, opportunities to act independently may be limited. With the rapid development of the Internet, a relatively freedom atmosphere allows people to express their thoughts and emotions without the threat of repercussion. According to the theory of self-determination, when young people’s autonomy needs are satisfied through the network, they will continue to pursue this activity. This study showed that there was a direct prediction effect between satisfaction of online autonomy need and self-disclosure. Interestingly, the impact of online autonomy need satisfaction and self-disclosure on the mobile net was not mediated by exhibitionism. One plausible explanation was that the motivation of self-disclosure online also included other motivations such as entertainment and passing time (Hollenbaugh and Ferris, 2014), which should be the focus of future studies.

### The Moderation Effect of Narcissism for the Relationship between Online Competence and Relatedness Psychological Needs Satisfaction and Exhibitionism

In this study, we explored whether narcissistic personality trait had a moderating effect on the front path from online competence and relatedness needs satisfaction on exhibitionism. The results of the study proved that the main effect of narcissistic personality trait on exhibitionism was not significant, inconsistent with previous research (Wang and Stefanone, 2013). However, further analysis showed that the interaction effect of narcissism and both competence and relatedness needs satisfaction could significantly and positively predict exhibitionism, and further explained that all the factors and conditions would not work alone, but work interactively. The impact mechanism of online basic psychological need satisfaction on self-disclosure on the mobile net is a complex integrated process.

As motivations are driving forces and psychological dispositions reinforce certain behavior to gratifying desires, different people with varying psychological and emotional states are motivated by different needs, which could be gratified in numerous ways via engagement with social media (Nabi et al., 2006). It is likely that different users access to mobile net for different underlying needs and that these needs may be associated with different types of motivations. People with a high level of narcissism have a strong desire to gain attention and approval of others. Higher degrees of competence need satisfaction from the network make narcissistic individuals feel more confident about their superiority, thus exhibiting more effort to get more people’s recognition and appreciation. In other words, our findings suggest that increased competence need satisfaction for people with a high level of narcissism did not necessarily increase the same extent of online exhibitionism to the same degree as it did for people with a low level of narcissism.

The moderating effect of narcissism on the relationship between online relatedness need satisfaction and exhibitionism was opposite to the above research results. When narcissistic individuals obtain a sense of belonging, they also tend to disclose more information online. Actually, narcissists required an audience to meet their constant need for admiration in order to enhance their feelings of self-importance instead of caring about other people’s feelings (Morf and Rhodewalt, 2001; Campbell and Foster, 2007; Carlson et al., 2011). It is possible that their feeling of belongingness is different from other people’s. They are usually not interested in forming strong interpersonal relationships but rather in establishing superficial weak connections (and they are also skilled at initiating them; Campbell and Foster, 2002). If a narcissistic individual feels very connected to others but not very competent, then he or she should experience a more pressing desire to become more competent than to become more connected. In contrast, for average people, one’s perceived online relationship formation was a stronger predictor of exhibitionism than for narcissistic individuals. One possible explanation was that adolescents adopt online exhibition as strategies to enhance online friendships resulting from their positive attitude toward online relationship formation.

### Implications and Suggestions for Future Research

Extending previous research, our findings provide empirical support for SDT in the context of the mobile net. The present study validated the influence mechanism of online basic psychological need satisfaction on self-discloser on the mobile net. It is helpful to improve our understanding of the self-disclosure behavior of adolescents.

First, these research results have some practical implications. On the one hand, Internet usage such as self-disclosure online affords opportunities to satisfy the basic psychological needs of young people; on the other hand, teenagers are eager to achieve personal perfection and independence, establish intimacy outside of their families, and develop social-emotional relationships. When they obtain a sense of competence, belongingness, and autonomy through disclosing private information on the Internet, they can become obsessed with network behavior. Due to a deficiency of self-control, young people might be caught in a vicious cycle. At present, how to help adolescents take advantage of self-disclosure behavior instead of becoming addicted to it has already become a research topic worthy of urgent attention.

Second, self-disclosure online preference in adolescents reflects a thought-provoking social phenomenon that is currently quite common in China. Young people may not have the chance to express their opinions freely and to be recognized and appreciated by the external world. In the real world, parents should affirm their teenagers’ strong points and good qualities through approaches such as praising and encouraging them. Teachers in the learning process should also pay attention to encouraging and inspiring students’ interest in study, and in the process of teaching, give classmates encouragement and praise, avoid fatigue, and help more teenagers to build confidence.

There are some limitations to this research that need to be improved in a future study. First, this study did not take the basic psychological need satisfaction in daily life into account. The basic psychological need satisfaction in daily life is one of the potential impact factors of self-disclosure on the mobile net. To a certain extent, it is worthwhile to explore the relationship among online and offline psychological need satisfaction and self-disclosure online. This research will focus on this relationship in the following study. Second, the method adopted in this study mainly includes questionnaire survey method. In view of the popularity of Social media like Facebook, Twitter, and blogs among young people, future study should be more concerned about the objective behavior of the self-disclosure online such as amount, breadth and depth through the big data analytics. In addition, it is helpful to systematically and thoroughly understand the continuous process and the rule of quantitative change and qualitative change among students by tracking studies. It would be helpful to explore the dynamic relationship between psychological need satisfaction and network behavior over a longer time period.

## Author Contributions

Conception and design of the study: YL, RL. Collection, analysis and interpretation of data: YL, RL, JW. Drafting the article: YL, RL. Revising the article critically: YL, RL, YD, RZ, and LX.

## Conflict of Interest Statement

The authors declare that the research was conducted in the absence of any commercial or financial relationships that could be construed as a potential conflict of interest.

## Supplementary Material

DATA SHEET 1 **Online basic psychological need satisfaction+exhibitionism+narcissism+self-disclosure questionnaire**.
