# Supplementary material for: How Online Basic Psychological Need Satisfaction Influences Self-Disclosure Online among Chinese Adolescents: Moderated Mediation Effect of Exhibitionism and Narcissism
Source: Front Psychol. 2016 Aug 26;7:1279. doi: 10.3389/fpsyg.2016.01279 (PMC5000554; doi:10.3389/fpsyg.2016.01279)
Supplement: Supplementary file 2 [file Data_Sheet_2.DOCX]

**Appendix**

English version questionnaire

·Online basic psychological need satisfaction questionnaire·

**Instruction:**

We sincerely thank you for participating in our survey. Please answer the following questions and tick the appropriate number according to your actual situation when accessing the internet through smart phone. Do not omit items. We appreciate your cooperation and support.

|  | **Items** | Strongly  Disagree | Moderately  Disagree | Slightly disagree | Neutral | | Slightly agree | | | | Moderately agree | | | Strongly  Agree |
| --- | --- | --- | --- | --- | --- | --- | --- | --- | --- | --- | --- | --- | --- | --- |
| 1 | I can decide which activities I want to do on the mobile net. | 1 | 2 | 3 | 4 | | | | 5 | | 6 | | | 7 |
| 2 | When I was on the mobile net, I feel I was supported by others online | 1 | 2 | 3 | 4 | | | | 5 | | 6 | | | 7 |
| 3 | I used the mobile net because I want to. | 1 | 2 | 3 | 4 | | | | 5 | | 6 | | | 7 |
| 4 | When I have participated in the mobile net for a while, I feel pretty competent. | 1 | 2 | 3 | 4 | | | | 5 | | 6 | | | 7 |
| 5 | I have a say regarding what kinds of thing I want to do on the mobile net. | 1 | 2 | 3 | 4 | | | | 5 | | 6 | | | 7 |
|  |  |  |  |  | |  | | |  |  | | |  | |
| 6 | I am pretty skilled on the mobile net. | 1 | 2 | 3 | | 4 | | 5 | | | | 6 | | 7 |
| 7 | When I was on the mobile net, I feel I was understood by others online. | 1 | 2 | 3 | | 4 | | 5 | | | | 6 | | 7 |
| 8 | I think I am pretty good at using the mobile net. | 1 | 2 | 3 | | 4 | | 5 | | | | 6 | | 7 |
| 9 | When I was on the mobile net, I feel I was listened to by others online. | 1 | 2 | 3 | | 4 | | 5 | | | | 6 | | 7 |
| 10 | I am satisfied with my performance on the mobile net. | 1 | 2 | 3 | | 4 | | 5 | | | | 6 | | 7 |
|  |  |  |  |  | |  | |  | |  | | |  | |
| 11 | I felt a certain freedom of action when I used the mobile Internet | 1 | 2 | 3 | | 4 | | 5 | | 6 | | | 7 | |
| 12 | When I was on the mobile net, I feel I was valued to others online. | 1 | 2 | 3 | | 4 | | 5 | | 6 | | | 7 | |

·Exhibitionism questionnaire (items 1-6) ·

·self-disclosure on the mobile net questionnaire (items 7-14) ·

**Instruction:**

We sincerely thank you for participating in our survey. Please answer the following questions and tick the appropriate number according to your actual situation when accessing the internet through smart phone. Do not omit items. We appreciate your cooperation and support.

|  | **Items** | Strongly disagreed | Disagree | Neutral | Agree | Strongly agree |
| --- | --- | --- | --- | --- | --- | --- |
| 1 | If no one can see, I'm not going to publish content on the mobile net | 1 | 2 | 3 | 4 | 5 |
| 2 | I check in because I like when people read things about me. | 1 | 2 | 3 | 4 | 5 |
| 3 | To get attention. | 1 | 2 | 3 | 4 | 5 |
| 4 | Because my posts make me cool among my peers. | 1 | 2 | 3 | 4 | 5 |
| 5 | I expect friends to ‘‘like’’ or leave comments on my status on the mobile net. | 1 | 2 | 3 | 4 | 5 |
| 6 | To gain fame or notoriety. | 1 | 2 | 3 | 4 | 5 |
| 7 | On the mobile Internet I talk about different topics more easily than during a face-to-face encounter. | 1 | 2 | 3 | 4 | 5 |
| 8 | On the mobile Internet I more easily change topics than in a face-to-face encounter. | 1 | 2 | 3 | 4 | 5 |
| 9 | On the mobile Internet, I can laugh more easily with others than in a face-to-face encounter. | 1 | 2 | 3 | 4 | 5 |
| 10 | On the mobile Internet, I can make others laugh more easily than in a face to-face encounter. | 1 | 2 | 3 | 4 | 5 |
| 11 | On the mobile Internet, I talk more easily about my inner feelings than in a face-to-face encounter. | 1 | 2 | 3 | 4 | 5 |
|  |  | |  |  |  |  |
| 12 | On the mobile Internet, I talk more easily about being in love than in a face-to-face encounter. | 1 | 2 | 3 | 4 | 5 |
| 13 | On the mobile Internet, I talk more easily about my worries than in a face-to-face encounter. | 1 | 2 | 3 | 4 | 5 |
| 14 | On the mobile Internet, I talk more easily about my secrets than in a face-to-face encounter. | 1 | 2 | 3 | 4 | 5 |

**Instruction:**

Please answer the following questions and tick the appropriate number according to the actual situation.

|  | **Items** | **Strongly**  **Disagree** | **Disagree** | **Neutral** | **Agree** | | **Strongly agree** | |
| --- | --- | --- | --- | --- | --- | --- | --- | --- |
| 1 | People see me as a natural leader. | 1 | 2 | 3 | | 4 | | 5 |
| 2 | I hate being the center of attention. | 1 | 2 | 3 | | 4 | | 5 |
| 3 | Many group activities tend to be dull without me. | 1 | 2 | 3 | | 4 | | 5 |
| 4 | I know that I am special because everyone keeps telling me so. | 1 | 2 | 3 | | 4 | | 5 |
| 5 | I like to get acquainted with important people. | 1 | 2 | 3 | | 4 | | 5 |
|  |  |  |  |  | |  | |  |
| 6 | I feel embarrassed if someone compliments me. | 1 | 2 | 3 | | 4 | | 5 |
| 7 | I have been compared to famous people. | 1 | 2 | 3 | | 4 | | 5 |
| 8 | I am an average person. | 1 | 2 | 3 | | 4 | | 5 |
| 9 | I insist on getting the respect I deserve. | 1 | 2 | 3 | | 4 | | 5 |
